# Supplementary material for: Coral reef fish assemblages exhibit signs of depletion in two protected areas from the eastern of Los Canarreos archipelago (Cuba, Caribbean Sea)
Source: PeerJ. 2022 Oct 14;10:e14229. doi: 10.7717/peerj.14229 (PMC9575676; doi:10.7717/peerj.14229)
Supplement: Supplemental Information 2 [file peerj-10-14229-s002.docx]

**Table S1.** Species recorded during the fish sampling in coral reefs of the Cayo Campos-Cayo Rosario Fauna Refuge, the Cayo Largo Ecological Reserve and contiguous non-protected sites. Functional classification is based on feeding habits and functional role (combination of feeding habits, mobility and size). Taxonomic classification is based on FishBase (Froese & Pauly, 2020), and functional classification in the criteria of Micheli et al. (2014) and the information available in FishBase (Froese & Pauly, 2020).

|  | **FAMILY** | **SPECIES** | **TROPHIC GROUP** | **FUNCTIONAL GROUP** |
| --- | --- | --- | --- | --- |
| **Class Elasmobranchii** |  |  |  |  |
| Order Myliobatiformes |  |  |  |  |
|  | Myliobatidae | *Aetobatus narinari* | Invertebrate feeders | Pelagic macroinvertivores |
|  | Dasyatidae | *Hypanus americanus* | Invertebrate feeders | Roving sand macroinvertivores |
| Order Orectolobiformes |  |  |  |  |
|  | Ginglymostomatidae | *Ginglymostoma cirratum* | Invertebrate feeders/piscivores | Midwater predators |
| **Class Actinopterygii** |  |  |  |  |
| Order Beryciformes |  |  |  |  |
|  | Holocentridae | *Holocentrus adscensionis* | Invertebrate feeders | Roving macroinvertivores |
|  | Holocentridae | *Holocentrus rufus* | Invertebrate feeders | Roving macroinvertivores |
|  | Holocentridae | *Myripristis jacobus* | Planktivores | Roving planktivores |
|  | Holocentridae | *Neoniphon marianus* | Invertebrate feeders | Small roving macroinvertivores |
| Order Syngnathiformes |  |  |  |  |
|  | Aulostomidae | *Aulostomus maculatus* | Piscivores | Roving piscivores |
| Order Scorpaeniformes |  |  |  |  |
|  | Scorpaenidae | *Pterois* sp*.* | Invertebrate feeders/piscivores | Cryptic predators |
| Order Perciformes |  |  |  |  |
|  | Serranidae | *Cephalopholis cruentata* | Piscivores | Roving piscivores |
|  | Serranidae | *Cephalopholis fulva* | Piscivores | Roving piscivores |
|  | Serranidae | *Epinephelus guttatus* | Invertebrate feeders/piscivores | Roving predators |
|  | Serranidae | *Epinephelus striatus* | Invertebrate feeders/piscivores | Roving predators |
|  | Serranidae | *Hypoplectrus indigo* | Invertebrate feeders | Small cryptic macroinvertivores |
|  | Serranidae | *Mycteroperca tigris* | Piscivores | Roving piscivores |
|  | Serranidae | *Mycteroperca venenosa* | Piscivores | Roving piscivores |
|  | Grammatidae | *Gramma loreto* | Invertebrate feeders | Cryptic microinvertivores |
|  | Malacanthidae | *Malacanthus plumieri* | Invertebrate feeders | Cryptic sand macroinvertivores |
|  | Carangidae | *Carangoides bartholomaei* | Piscivores | Midwater piscivores |
|  | Carangidae | *Caranx latus* | Piscivores | Pelagic piscivores |
|  | Carangidae | *Caranx ruber* | Piscivores | Midwater piscivores |
|  | Lutjanidae | *Lutjanus analis* | Invertebrate feeders/piscivores | Roving predators |
|  | Lutjanidae | *Lutjanus apodus* | Invertebrate feeders/piscivores | Roving predators |
|  | Lutjanidae | *Lutjanus cyanopterus* | Invertebrate feeders/piscivores | Roving predators |
|  | Lutjanidae | *Lutjanus griseus* | Invertebrate feeders/piscivores | Roving predators |
|  | Lutjanidae | *Lutjanus jocu* | Invertebrate feeders/piscivores | Roving predators |
|  | Lutjanidae | *Lutjanus mahogoni* | Invertebrate feeders/piscivores | Roving predators |
|  | Lutjanidae | *Ocyurus chrysurus* | Invertebrate feeders/piscivores | Midwater predators |
|  | Haemulidae | *Anisotremus virginicus* | Invertebrate feeders | Roving macroinvertivores |
|  | Haemulidae | *Haemulon album* | Invertebrate feeders | Roving macroinvertivores |
|  | Haemulidae | *Haemulon carbonarium* | Invertebrate feeders | Roving macroinvertivores |
|  | Haemulidae | *Haemulon chrysargyreum* | Invertebrate feeders | Small roving macroinvertivores |
|  | Haemulidae | *Haemulon flavolineatum* | Invertebrate feeders | Roving macroinvertivores |
|  | Haemulidae | *Haemulon parra* | Invertebrate feeders | Roving macroinvertivores |
|  | Haemulidae | *Haemulon plumierii* | Invertebrate feeders | Roving macroinvertivores |
|  | Haemulidae | *Haemulon sciurus* | Invertebrate feeders | Roving macroinvertivores |
|  | Haemulidae | *Haemulon vittatum* | Planktivores | Pelagic planktivores |
|  | Sparidae | *Calamus* sp. | Invertebrate feeders | Roving macroinvertivores |
|  | Mullidae | *Mulloidichthys martinicus* | Invertebrate feeders | Roving macroinvertivores |
|  | Mullidae | *Pseudupeneus maculatus* | Invertebrate feeders | Microinvertivores |
|  | Kyphosidae | *Kyphosus* sp*.* | Omnivores | Midwater omnivores |
|  | Chaetodontidae | *Chaetodon capistratus* | Invertebrate feeders | Roving sessile invertivores |
|  | Chaetodontidae | *Chaetodon ocellatus* | Invertebrate feeders | Microinvertivores |
|  | Chaetodontidae | *Chaetodon striatus* | Invertebrate feeders | Microinvertivores |
|  | Pomacanthidae | *Holacanthus ciliaris* | Invertebrate feeders | Cryptic sessile invertivores |
|  | Pomacanthidae | *Holacanthus tricolor* | Invertebrate feeders | Roving sessile invertivores |
|  | Pomacanthidae | *Pomacanthus arcuatus* | Invertebrate feeders | Roving sessile invertivores |
|  | Pomacanthidae | *Pomacanthus paru* | Invertebrate feeders | Roving sessile invertivores |
|  | Pomacentridae | *Abudefduf saxatilis* | Invertebrate feeders | Cryptic microinvertivores |
|  | Pomacentridae | *Chromis cyanea* | Planktivores | Cryptic planktivores |
|  | Pomacentridae | *Chromis multilineata* | Planktivores | Cryptic planktivores |
|  | Pomacentridae | *Microspathodon chrysurus* | Herbivores | Cryptic grazers |
|  | Pomacentridae | *Stegastes adustus* | Herbivores | Cryptic grazers |
|  | Pomacentridae | *Stegastes diencaeus* | Herbivores | Cryptic grazers |
|  | Pomacentridae | *Stegastes leucostictus* | Herbivores | Roving grazers |
|  | Pomacentridae | *Stegastes partitus* | Herbivores | Cryptic grazers |
|  | Pomacentridae | *Stegastes planifrons* | Herbivores | Roving browsers |
|  | Pomacentridae | *Stegastes variabilis* | Herbivores | Cryptic grazers |
|  | Labridae | *Bodianus rufus* | Invertebrate feeders | Roving macroinvertivores |
|  | Labridae | *Clepticus parrae* | Planktivores | Midwater planktivores |
|  | Labridae | *Halichoeres bivittatus* | Invertebrate feeders | Small roving macroinvertivores |
|  | Labridae | *Halichoeres garnoti* | Invertebrate feeders | Small roving macroinvertivores |
|  | Labridae | *Halichoeres maculipinna* | Invertebrate feeders | Microinvertivores |
|  | Labridae | *Halichoeres poeyi* | Invertebrate feeders | Small roving macroinvertivores |
|  | Labridae | *Halichoeres radiatus* | Invertebrate feeders | Roving macroinvertivores |
|  | Labridae | *Lachnolaimus maximus* | Invertebrate feeders | Roving macroinvertivores |
|  | Labridae | *Thalassoma bifasciatum* | Planktivores | Roving planktivores |
|  | Scaridae | *Scarus guacamaia* | Herbivores | Large roving browsers |
|  | Scaridae | *Scarus iseri* | Herbivores | Roving grazers |
|  | Scaridae | *Scarus taeniopterus* | Herbivores | Roving browsers |
|  | Scaridae | *Scarus vetula* | Herbivores | Roving browsers |
|  | Scaridae | *Sparisoma aurofrenatum* | Herbivores | Roving grazers |
|  | Scaridae | *Sparisoma chrysopterum* | Herbivores | Roving browsers |
|  | Scaridae | *Sparisoma rubripinne* | Herbivores | Roving browsers |
|  | Scaridae | *Sparisoma viride* | Herbivores | Roving browsers |
|  | Acanthuridae | *Acanthurus chirurgus* | Herbivores | Roving grazers |
|  | Acanthuridae | *Acanthurus coeruleus* | Herbivores | Roving grazers |
|  | Acanthuridae | *Acanthurus tractus/ Acanthurus bahianus* | Herbivores | Roving grazers |
|  | Sphyraenidae | *Sphyraena barracuda* | Piscivores | Pelagic piscivores |
| Order Tetraodontiformes |  |  |  |  |
|  | Balistidae | *Balistes vetula* | Invertebrate feeders | Roving macroinvertivores |
|  | Balistidae | *Canthidermis sufflamen* | Planktivores | Midwater planktivores |
|  | Balistidae | *Melichthys niger* | Planktivores | Midwater planktivores |
|  | Balistidae | *Xanthichthys ringens* | Invertebrate feeders | Midwater macroinvertivores |
|  | Diodontidae | *Diodon hystrix* | Invertebrate feeders | Cryptic macroinvertivores |
