# Supplementary figures and images for: Coral reef fish assemblages exhibit signs of depletion in two protected areas from the eastern of Los Canarreos archipelago (Cuba, Caribbean Sea)
Source: PeerJ. 2022 Oct 14;10:e14229. doi: 10.7717/peerj.14229 (PMC9575676; doi:10.7717/peerj.14229)

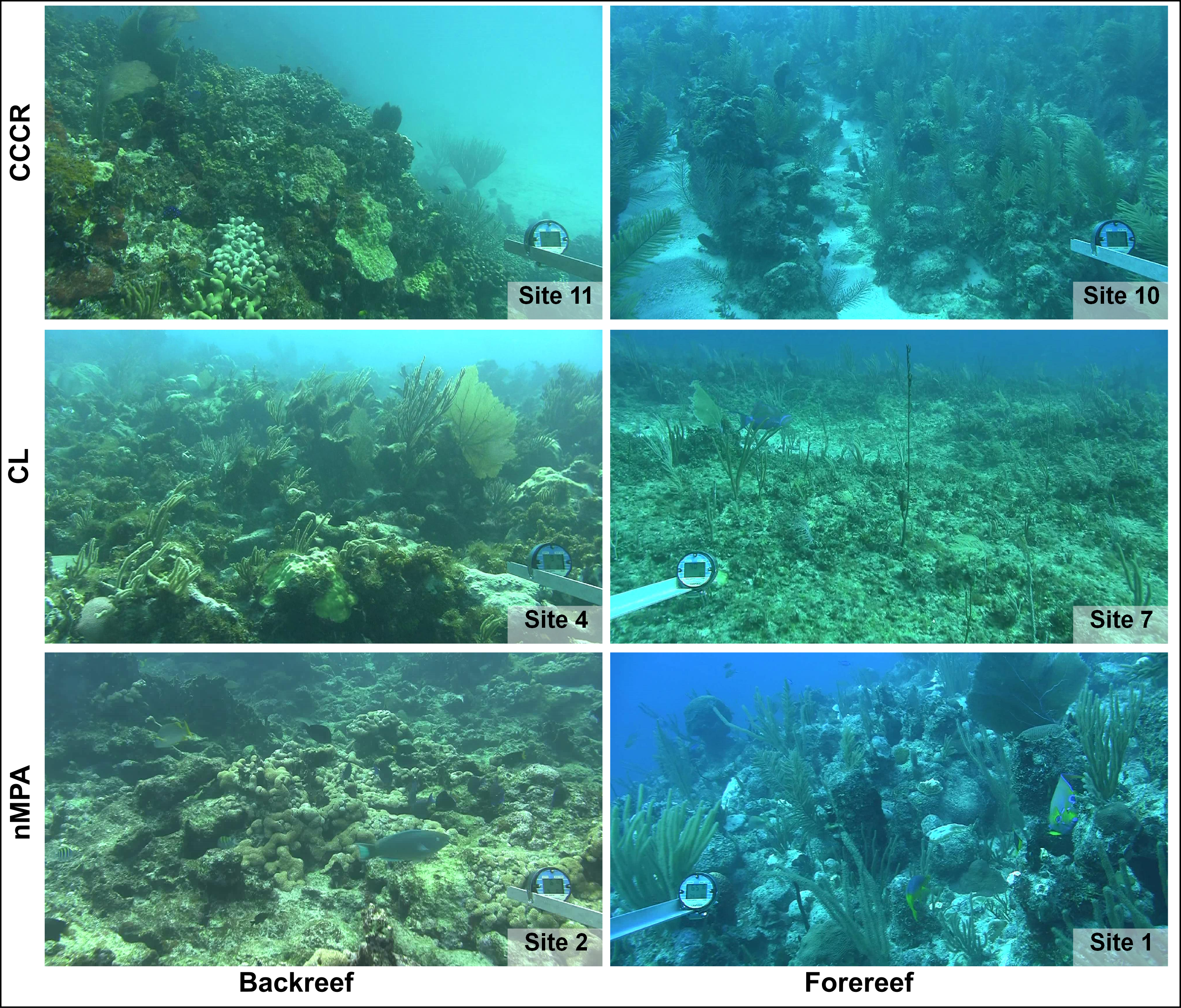

Supplement: Supplemental Information 3 — Note that each picture has the number of the surveyed site. [file peerj-10-14229-s003.png]

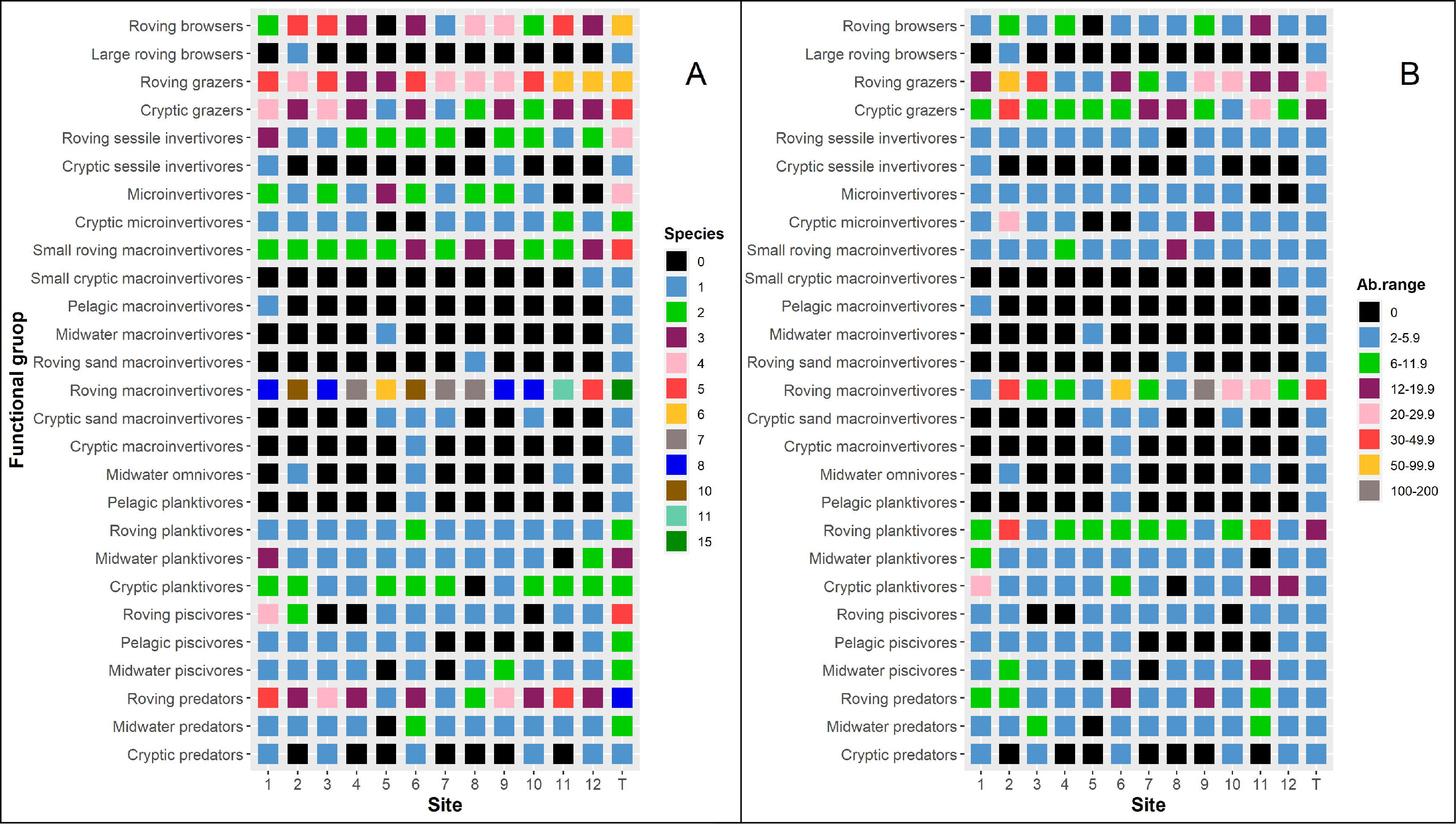

Supplement: Supplemental Information 4 — Functional redundancy is given as cumulative number of species per functional groups along each site (1–12) and along the study (T). Averaged abundance (individuals per 125 m2) of functional groups is given also per site (1–12) and along the study (T) scaled by range (Ab.range). [file peerj-10-14229-s004.png]
